# Human Infection with MERS Coronavirus after Exposure to Infected Camels, Saudi Arabia, 2013

**DOI:** 10.3201/eid2006.140402

**Published:** 2014-06

**Authors:** Ziad A. Memish, Matthew Cotten, Benjamin Meyer, Simon J. Watson, Abdullah J. Alsahafi, Abdullah A. Al Rabeeah, Victor Max Corman, Andrea Sieberg, Hatem Q. Makhdoom, Abdullah Assiri, Malaki Al Masri, Souhaib Aldabbagh, Berend-Jan Bosch, Martin Beer, Marcel A. Müller, Paul Kellam, Christian Drosten

**Affiliations:** Al Faisal University, Riyadh, Saudi Arabia (Z.A. Memish);; Global Centre for Mass Gatherings Medicine, Riyadh (Z.A. Memish, A.A. Al Rabeeh);; Ministry of Health, Riyadh (Z.A. Memish, A.A. Al Rabeeh, A. Assiri, M. Al Masri);; Wellcome Trust Sanger Institute, Hinxton, UK (M. Cotten, S.J. Watson, P. Kellam);; University of Bonn Medical Centre Institute of Virology, Bonn, Germany (B. Meyer, V.M. Corman, A. Sieberg, S. Aldabbagh, M.A. Müller, C. Drosten);; Regional Health Directorate, Jeddah, Saudi Arabia (A.J. Alsahafi);; Jeddah Regional Laboratory, Jeddah (Hatem Q. Makhdoom);; Utrecht University, the Netherlands (B.-J. Bosch);; Friedrich-Loeffler-Institut, Institute for Virus Diagnostics, Greifswald–Insel Riems, Germany (M. Beer);; University College London, London, UK (P. Kellam)

**Keywords:** MERS, coronavirus, viruses, dromedary camels, Saudi Arabia, Middle East respiratory syndrome, respiratory infections

## Abstract

We investigated a case of human infection with Middle East respiratory syndrome coronavirus (MERS-CoV) after exposure to infected camels. Analysis of the whole human-derived virus and 15% of the camel-derived virus sequence yielded nucleotide polymorphism signatures suggestive of cross-species transmission. Camels may act as a direct source of human MERS-CoV infection.

Middle East respiratory syndrome coronavirus (MERS-CoV) was identified in 2012 in a cell culture taken from a patient who died of pneumonia in Saudi Arabia (*1*). Since 2012, at least 187 laboratory-confirmed human cases of MERS-CoV infection, most resulting in respiratory tract illness, have been reported to the World Health Organization; 97 of these cases were fatal. Known cases have been directly or indirectly linked to countries in the Arabian Peninsula (*2*). Dromedary camels across and beyond the region show high rates of antibodies against MERS-CoV (*3*–*7*), and viral RNA has been detected in camels in different countries (*8*,*9*). In 1 instance, a camel and 2 humans caring for the camel were found to be infected with viruses that were highly similar but distinct within 4,395 nt of the camel-derived virus sequence, including several phylogenetically informative nucleotide changes (*10*). To investigate possible camel–human virus transmission, we analyzed an infection with MERS-CoV in a man after he had contact with an infected camel.

## The Study

On November 3, 2013, the Ministry of Health of Saudi Arabia was notified of a suspected case of MERS-CoV infection in a 43-year-old male patient at King Abdulaziz University Hospital in Jeddah. The patient had cared for ill camels in his herd of 9 animals starting in early October, when the patient noted respiratory signs of illness with nasal discharge in several animals; he continued caring for the sick animals until October 27, the day of onset of his own illness. The patient cared for the animals for ≈3 hours per day 3 days per week, applying herbal remedies to the animals’ snouts and nostrils. He did not clean the stables or milk the animals, but he routinely consumed raw, unpasteurized camel milk from the herd.

Presence of MERS-CoV RNA in the patient was confirmed at Jeddah Regional Laboratory by using reverse transcription PCR (RT-PCR) targeting the *upE* and *orfA* gene fragments (*11*,*12*). Respiratory swab specimens yielded detectable signal after 28 RT-PCR cycles, indicative of an approximate viral load of 350,000 RNA copies per sample. A nearly complete viral genome was obtained (Jeddah_1_2013; GenBank accession no. KJ556336), confirming the presence of a typical MERS-CoV whose closest relatives were in the Riyadh_3 clade, as defined in (*2*) (phylogeny shown in online Technical Appendix Figure 1, wwwnc.cdc.gov/EID/article/20/6/14-0402-Techapp1.pdf).

To identify potential sources of infection, on November 9, the Ministry of Health investigated 5 close household contacts and the animal attendant on a farm owned by the patient. Nasopharyngeal swab samples were taken and tested at Jeddah Regional Laboratory by using RT-PCR. Deep nasal swab specimens were taken on the same day from 3 of the 9 camels at the farm. Testing of all samples by RT-PCR using the *upE* assay (*11*,*12*) did not detect MERS-CoV RNA in any of the human patients, but 1 of the 3 camels (camel G) tested positive (cycle threshold [C_t_] = 33). On November 13, nasal swab samples were obtained from all 9 animals. *upE* RT-PCR results were positive for camel G (C_t_ = 38) and a second camel (camel B; C_t_ = 39).

Samples from November 13 and a small remaining amount of RNA extract from camel G from November 9 were sent to the Sanger Institute in Cambridge, UK, and confirmation of reactivity (C_t_ ≈ 38) was obtained for pooled samples with the *upE* assay from camel G but not for camel B. The result for camel G was confirmed at the Institute of Virology in Bonn, Germany, for the same samples by using real-time RT-PCRs targeting the *upE* and 1A diagnostic target regions.

A sequence of ≈15% of the camel-derived genome was determined from 8 RT-PCR fragments, 2 of them partially overlapping (4,608 nt total) (*13*). Phylogenetic analyses supported the conclusion that transmission occurred between camel and patient, but no direction was implied (e.g, camel to human vs. human to camel; online Technical Appendix Figure 1). The human- and camel-derived sequences shared a signature of single nucleotide polymorphisms that occurred in no other known MERS-CoV sequences (Figure). Single nucleotide exchanges occurred at nt positions 21945 and 29662; these exchanges might have arisen during virus transmission, as described (*10*). However, because of the low RNA concentration in the samples, reamplification of the material and investigation of possible PCR-based mutations could not be done.

**Figure:**
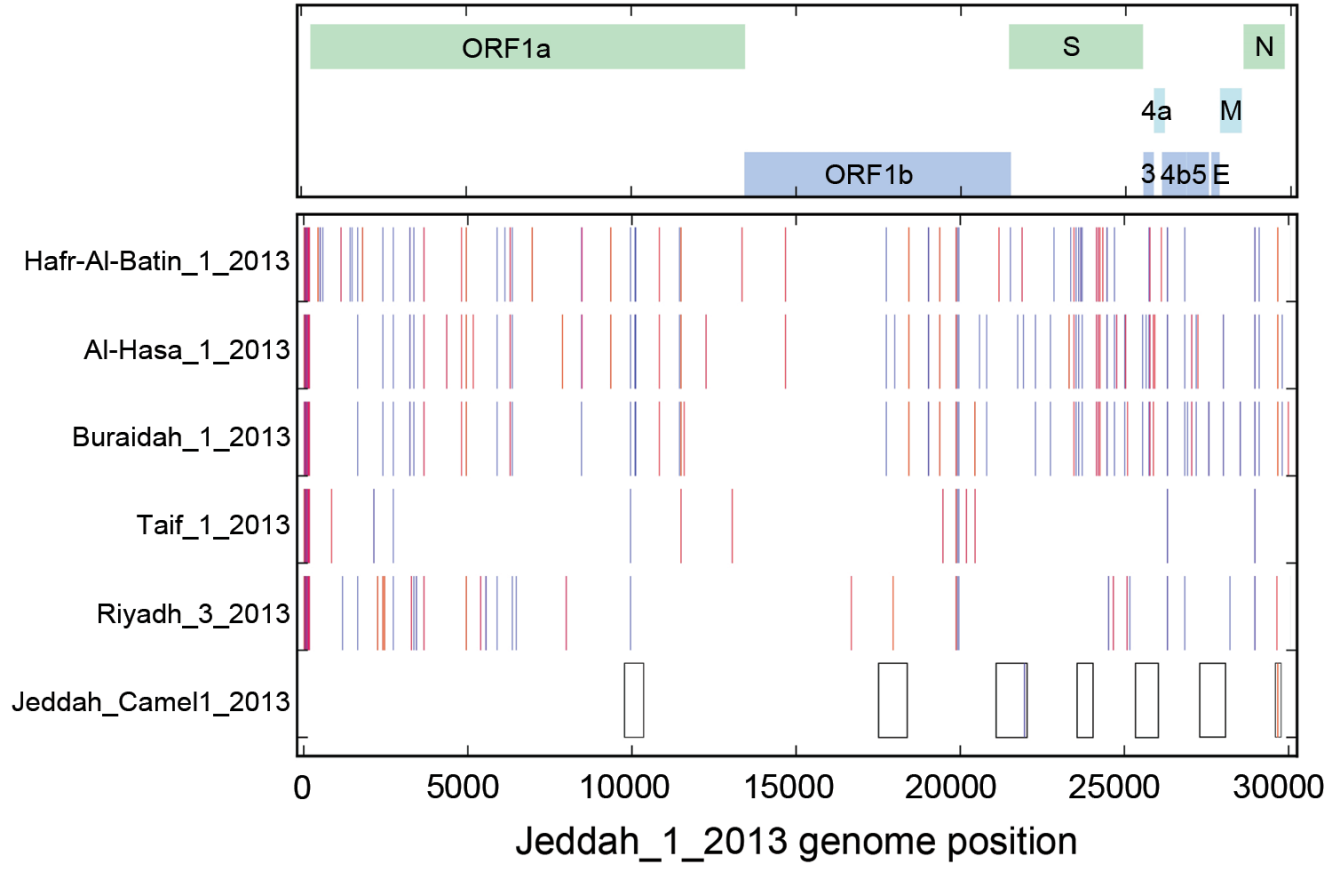
Direct comparison of the Middle East respiratory syndrome coronavirus (MERS-CoV) Jeddah_1_2013 genome sequence, Jeddah_ Camel1_2013 fragments (boxes at bottom), and representative genomes of other clade viruses: 2 additional genomes from the Riyadh_3 clade, Riyadh_3_2013 and Taif_1_2013; and representative genomes from the Al-Hasa and Hafr-Al-Batin_1 and Buraidah_1 clades. A map of the MERS-CoV genome with the major open reading frames (ORFs) indicated is shown at the top. Nucleotide differences for other genomes from Jeddah_1_2013 are shown by vertical colored bars: orange, change to A; red, change to T; blue, change to G; violet, change to C. Gaps in all full-genome sequences are indicated in gray. Positions according to the MERS-CoV genome EMC/2012: fragment 1, 9767–10354; fragment 2, 17507–18394; fragment 3, 21089–22046; fragment 4, 23569–24059; fragment 5, 25349–26056; fragment 6, 27276–28095; fragment 7, 29596–29757. The sequences reported here have been deposited in GenBank (accession nos. KJ556337–KJ556340; others are pending).

A serum sample taken from camel G during the initial investigation on November 9 was tested by recombinant immunofluorescence assay (IFA) as described (*6*,*14*) and showed reactivity to MERS-CoV (titer 320). To investigate signs of recent MERS-CoV infection in the group of camels, we obtained blood samples at short intervals from all 9 animals during November 14–December 9, 2013. All samples were tested by ELISA against recombinant MERS-CoV spike antigen domain S1 fused to human Fc fragment, using a formulation as described (*15*). Serum samples from all 9 animals showed reactivity to the MERS-CoV antigen; serum samples from control animals showed no reactivity (online Technical Appendix Figure 2, panel A). ELISA signals were constant over time in most animals, but a small, yet visible, change of signal over the observation period was noted for camels B and G. To clarify the reasons for this putative signal increase, the first and last serum samples (obtained on November 14 and December 9) from all animals were re-tested by IFA. As summarized in the Table, camels B and G showed 4-fold increases of titer for the paired first and last serum samples. In serologic tests that rely on 2-fold endpoint titrations, a titer increase >2 dilution steps is considered a significant sign of recent acute infection. For additional confirmation of rises of titer, sequential samples from camels B and G were compared with sequential samples from camels E and I by using IFA with endpoint titration. These data confirmed the increases of titers for camels B and G (online Technical Appendix Figure 2, panel B).

Because bovine CoV occurs in camels, we tested for antibodies against bovine CoV in camels B and G to exclude potential cross-reactions. Using IFA (*6*), we found no bovine CoV antibodies in camel G, but camel B showed rising bovine CoV antibody titers (Table). To obtain further differentiation, we performed neutralization assays against MERS-CoV and bovine CoV (*7*). Titers in serum samples from November 14 and December 9, respectively, were 160 and 320 for camel B and 160 and 160 for camel G. None of the animals showed serum neutralization against bovine CoV.

**Table:** Reciprocal immunofluorescence titers for MERS and bovine CoV in sequential serum samples from 9 camels, Jeddah, Saudi Arabia, 2013*

Camel	Age	Anti–MERS CoV titers			Anti–bovine CoV titers	
		Nov 14	Dec 9		Nov 14	Dec 9
A	13 y	40,960	81,920			
B	3 mo	640	2,560		320	1,280–2,560
C	12 y	20,480	20,480			
D	9 y	40,960	40,960			
E	13 y	5,120	5,120			
F	8 y	40,960	40,960			
G†	8 mo	640	2,560		<10	<10
H	8 mo	40,960	40,960			
I	2 y	5,120	5,120			

*MERS, Middle East respiratory syndrome; CoV, coronavirus. †For camel G, a sample taken on November 9 was also available and yielded an immunofluorescence titer of 320.

## Conclusions

These data add to recent findings showing high similarity of MERS-CoVs carried by humans and camels (*8*,*10*), supporting the hypothesis that human MERS-CoV infection may be acquired directly from camels. In addition, both animals that showed signs of recent infection were juvenile, which provides further support to previous findings that mainly young animals are infected by MERS-CoV (*7*,*8*). Given the synchronized parturition pattern of dromedary camels, with birthing in the winter months, an increase of epizootic activity might be expected after some latency during the first half of each year.

Our data provide particular insight into the timing of infections and transmission. Antibody titers rose and viral RNA concentrations were already on the decline in the camels while the patient was hospitalized with acute symptoms. Assuming a time before appearance of antibodies of 10–21 days, at least some of the camels would have been actively infected during middle to late October, when some animals showed signs of respiratory illness and the patient acquired his infection. Nevertheless, we cannot rule out other infectious causes of the animals’ upper respiratory signs. Also, because of the retrospective nature of this investigation, we cannot rule out the possibility of a third source of MERS-CoV infection for camels and humans.

Technical Appendix Maximum-clade credibility tree of 66 human Middle East respiratory syndrome coronavirus (MERS-CoV) genomes and 3 camel genomes or genome fragments and results of serologic screening of 9 dromedary camels from Jeddah, Saudi Arabia, 2013.

## References

[1] Zaki AM, van Boheemen S, Bestebroer TM, Osterhaus AD, Fouchier RA. Isolation of a novel coronavirus from a man with pneumonia in Saudi Arabia. N Engl J Med. 2012;367:1814–20. 10.1056/NEJMoa1211721 23075143

[2] Cotten M, Watson SJ, Zumla AI, Makhdoom HQ, Palser AL, Ong SH, Spread, circulation, and evolution of the middle East respiratory syndrome coronavirus. MBio. 2014;5:e01062–13. 10.1128/mBio.01062-13 24549846 PMC3944817

[3] Hemida MG, Perera RA, Wang P, Alhammadi MA, Siu LY, Li M, Middle East respiratory syndrome (MERS) coronavirus seroprevalence in domestic livestock in Saudi Arabia, 2010 to 2013. Euro Surveill. 2013;18:20659. 24342517 10.2807/1560-7917.es2013.18.50.20659

[4] Reusken CB, Ababneh M, Raj VS, Meyer B, Eljarah A, Abutarbush S, Middle East respiratory syndrome coronavirus (MERS-CoV) serology in major livestock species in an affected region in Jordan, June to September 2013. Euro Surveill. 2013;18:20662. 24342516 10.2807/1560-7917.es2013.18.50.20662

[5] Perera RA, Wang P, Gomaa MR, El-Shesheny R, Kandeil A, Bagato O, Seroepidemiology for MERS coronavirus using microneutralisation and pseudoparticle virus neutralisation assays reveal a high prevalence of antibody in dromedary camels in Egypt, June 2013. Euro Surveill. 2013;18:20574. 24079378 10.2807/1560-7917.es2013.18.36.20574

[6] Reusken CB, Haagmans BL, Muller MA, Gutierrez C, Godeke GJ, Meyer B, Middle East respiratory syndrome coronavirus neutralising serum antibodies in dromedary camels: a comparative serological study. Lancet Infect Dis. 2013;13:859–66. 10.1016/S1473-3099(13)70164-6 23933067 PMC7106530

[7] Meyer B, Müller MA, Corman VM, Reusken CBEM, Ritz D, Godeke G-D, Antibodies against MERS coronavirus in dromedary camels, United Arab Emirates, 2003 and 2013. Emerg Infect Dis. 2014;20:552–9. 10.3201/eid2004.131746 24655412 PMC3966379

[8] Alagaili AN, Briese T, Mishra N, Kapoor V, Sameroff SC, de Wit E, Middle East respiratory syndrome coronavirus infection in dromedary camels in Saudi Arabia. MBio. 2014;5:e00884–14. 10.1128/mBio.00884-14 24570370 PMC3940034

[9] Chu DKW, Poon LLM, Gomaa MM, Shehata MM, Perera RAPM, Zeid DAE, MERS coronaviruses in dromedary camels, Egypt. Emerg Infect Dis. 2014; [Epub ahead of print]. 10.3201/eid2006.140299 PMC4036765 24856660

[10] Haagmans BL, Al Dhahiry SH, Reusken CB, Raj VS, Galiano M, Myers R, Middle East respiratory syndrome coronavirus in dromedary camels: an outbreak investigation. Lancet Infect Dis. 2014;14:140–5. 10.1016/S1473-3099(13)70690-X 24355866 PMC7106553

[11] Corman VM, Eckerle I, Bleicker T, Zaki A, Landt O, Eschbach-Bludau M, Detection of a novel human coronavirus by real-time reverse-transcription polymerase chain reaction. Euro Surveill. 2012;17:20285. 23041020 10.2807/ese.17.39.20285-en

[12] Corman VM, Muller M, Costabel U, Timm J, Binger T, Meyer B, Assays for laboratory confirmation of novel human coronavirus (hCoV-EMC) infections. Euro Surveill. 2012;17:20334. 23231891 10.2807/ese.17.49.20334-en

[13] Cotten M, Lam TT, Watson SJ, Palser AL, Petrova V, Grant P, Full-genome deep sequencing and phylogenetic analysis of novel human betacoronavirus. Emerg Infect Dis. 2013;19:736–42. 10.3201/eid1905.130057 23693015 PMC3647518

[14] Buchholz U, Muller MA, Nitsche A, Sanewski A, Wevering N, Bauer-Balci T, Contact investigation of a case of human novel coronavirus infection treated in a German hospital, October–November 2012. Euro Surveill. 2013;18:20406. 23449231

[15] Raj VS, Mou H, Smits SL, Dekkers DH, Muller MA, Dijkman R, Dipeptidyl peptidase 4 is a functional receptor for the emerging human coronavirus-EMC. Nature. 2013;495:251–4. 10.1038/nature12005 23486063 PMC7095326

