# Supplementary material for: Human Infection with MERS Coronavirus after Exposure to Infected Camels, Saudi Arabia, 2013
Source: Emerg Infect Dis. 2014 Jun;20(6):1012–5. doi: 10.3201/eid2006.140402 (PMC4036761; doi:10.3201/eid2006.140402)
Supplement: Technical Appendix — Maximum-clade credibility tree of 66 human Middle East respiratory syndrome coronavirus (MERS-CoV) genomes and 3 camel genomes or genome fragments and results of serologic screening of 9 dromedary camels from Jeddah, Saudi Arabia, 2013. [file 14-0402-Techapp-s1.pdf]

Article DOI: http://dx.doi.org/10.3201/eid2006.140402

Human Infection with MERS Coronavirus after Exposure to Infected Camels, Saudi Arabia, 2013

Technical Appendix

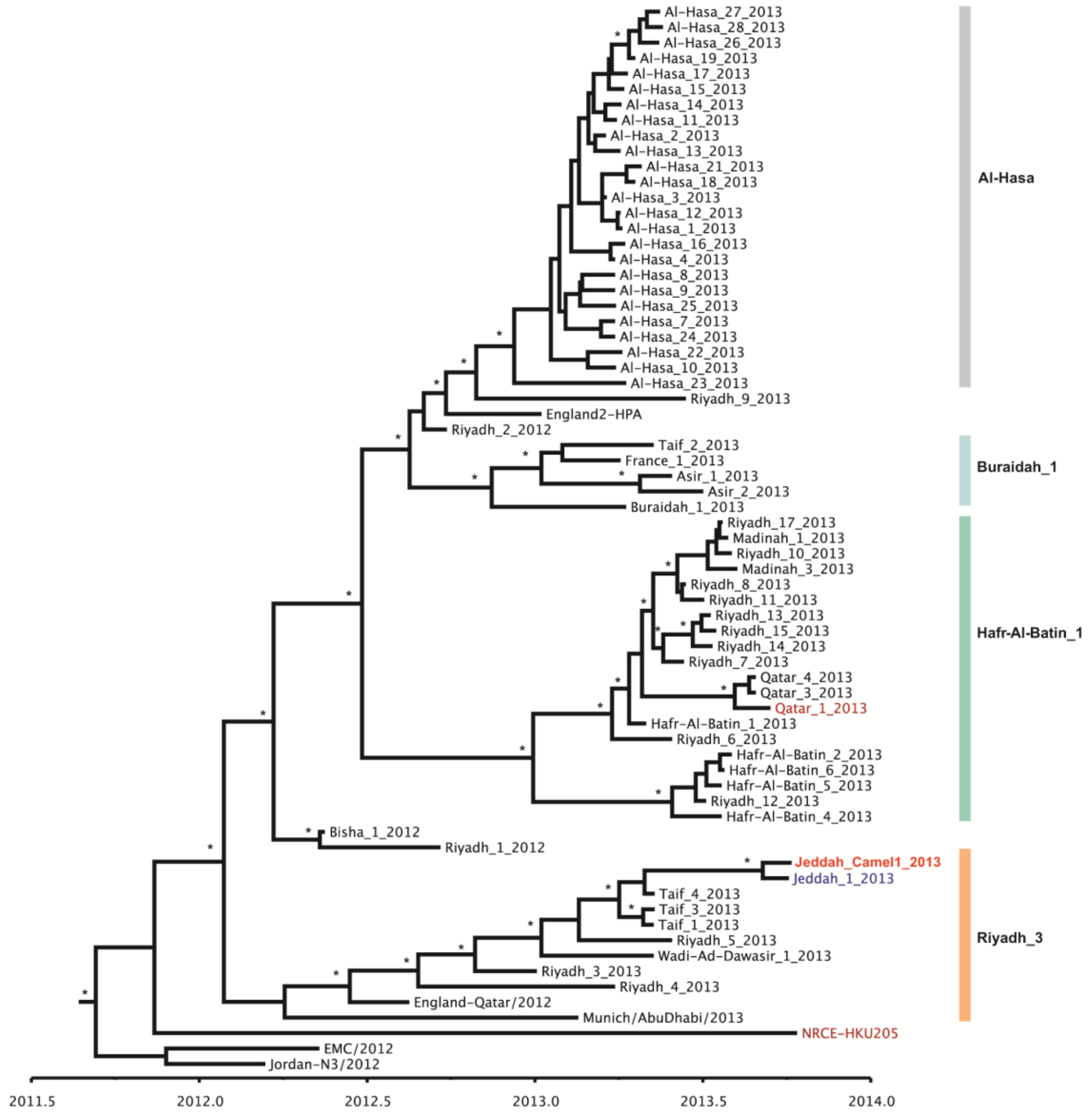

Technical Appendix Figure 1. Bayesian-inferred time-resolved maximum-clade credibility tree of 66 human MERS-CoV genomes and 3 camel genomes or genome-fragments. Highlighted in orange is the partial camel sequence generated in this study (Jeddah_Camel1_2013, GenBank accession number

pending), and in blue is the MERS-CoV genome from the camel owner (Jeddah_1_2013, GenBank accession number pending). Additional camel MERS-CoV sequences are indicated in dark red. Asterisks indicate nodes with a posterior probability greater than 0.9. The phylogeny was inferred using BEAST version 1.8.0 under an HKY+G substitution model, an uncorrelated lognormal molecular clock, and a GMRF skyride coalescent model. Trees were visualized and annotated using FigTree version 1.4.0 (http://tree.bio.ed.ac.uk/software/figtree/).

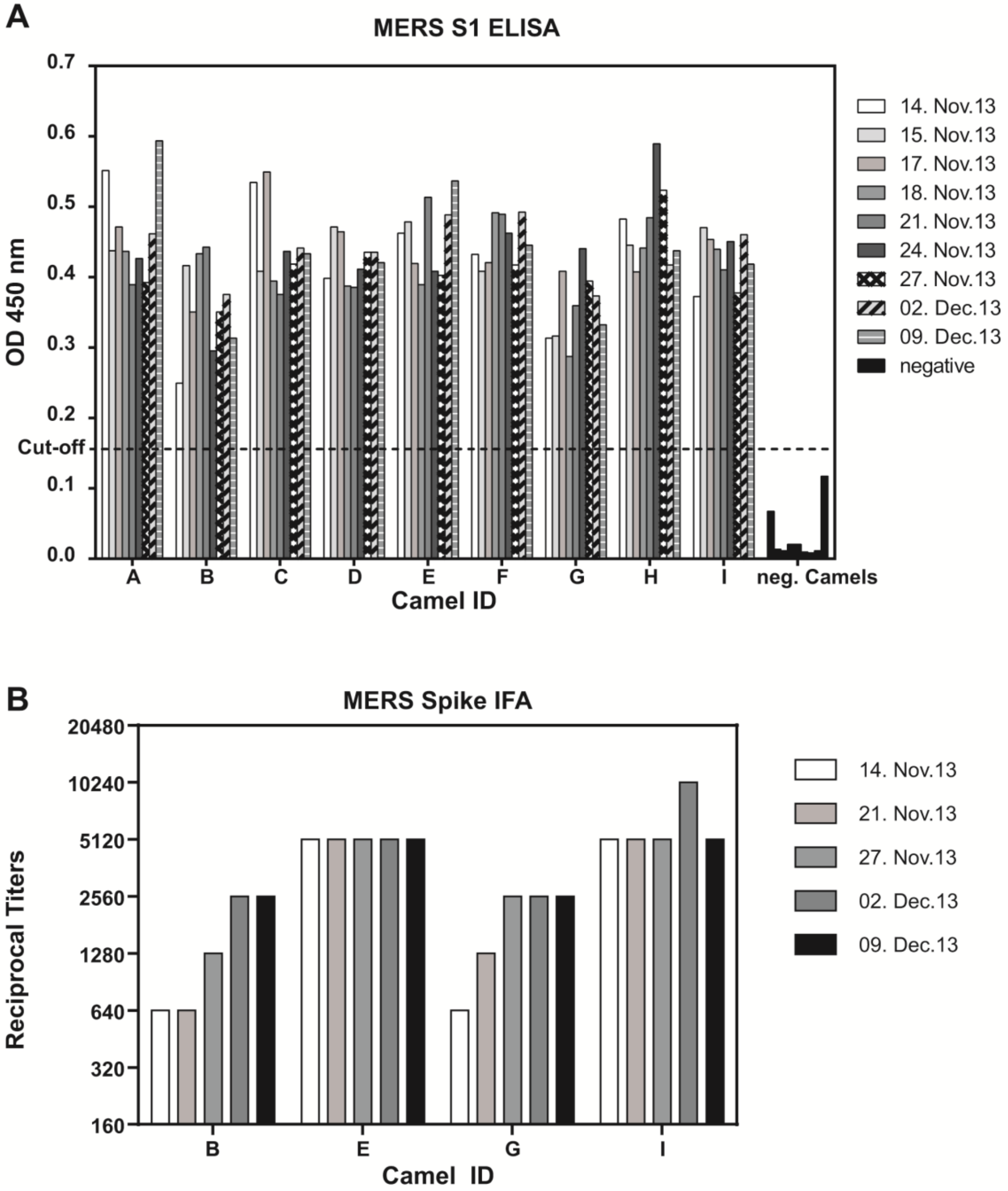

Technical Appendix Figure 2. Serologic screening of nine dromedary camels from Jeddah over a period of 4 weeks. A, Dromedary camel sera were tested by a MERS-CoV spike protein S1-subunit based

ELISA at a dilution of 1:100. Optical density was measured at 450 nm. The cutoff (dashed line) was set to three times the OD450 value of a selection of seronegative camel sera. Results from camel sera previously tested negative (*7*) are shown for comparison. B, Reciprocal endpoint titers as determined by recombinant immunofluorescence assay for animals with rise in titer (B, G) and animals with constant titer (E, I).
